# Transcoronary sinus repair of a severely dilated and tortuous right coronary artery fistulized to coronary sinus complicated with tricuspid valve insufficiency

**DOI:** 10.1186/s13019-022-02047-7

**Published:** 2022-12-14

**Authors:** Xuebiao Li, Jianfang Qian, Zhonghua Shen, Aiqiang Dong

**Affiliations:** 1 Cardiovascular Surgery, The Second Affiliated Hospital of Zhejiang University School of Medicine (SAHZU), No. 88 Jiefang Road, Hangzhou, Zhejiang Province China; 2 Center for Diagnosis and Treatment of Cardiovascular Disease, Hangzhou, Zhejiang Province China; 3 National Regional Center for Cardiovascular Disease, Hangzhou, China

**Keywords:** Coronary artery dilation, Coronary artery fistula, Tricuspid valve regurgitation, Adult congenital heart disease

## Abstract

**Background:**

Right coronary artery (RCA) fistulized to the coronary sinus is rare condition in adult cardiac anomalies, and the management and operative indication are controversial.

**Case presentation:**

We describe the case of a 45-year female patient who presented with exertional dyspnea, accompanied by intermitted lower limbs and facial edema. She was diagnosed with severe tricuspid regurgitation second to a severely dilated RCA fistulized to the coronary sinus. After multidisciplinary discussion, she underwent surgery through routine medium sternotomy, the right atrium was opened under cardiopulmonary bypass. The coronary arteriovenous fistula from the distal portion of RC to a severely enlarged coronary sinus was found. Trans-coronary sinus closure of the fistula was performed with continuous stitching and a tricuspid ring annuloplasty was done. The patient recovered uneventful post operation.

**Conclusion:**

According to current literatures, surgical treatment was adopted for this case, instead of endovascular intervention. The optimal approach for these cases should consider the heart’s anatomical characteristics. But we need to be aware of the occurrence of myocardial infarction and tricuspid regurgitation in the early and late stage after operation.

## Background

Coronary artery to coronary sinus fistula (CAF) is a relatively uncommon entity, reported 0.2–0.4% of all congenital heart diseases [1]. The pathological connection of the coronary artery into other cardiovascular structures often results in a marked dilation of the donor coronary artery leading volume overload or coronary artery flow stealing.

It could be discovered at any age, and most CAFs are small and present no signs and symptoms. But it could progressively enlarge with age and cause complications like heart failure, myocardial infarction, arrhythmias, infectious endocarditis, aneurysm formation, rupture. Based on limited literatures, the reasonable treatment and operative indication are controversial.

Here, we describe our case of a female who underwent successful surgical management of an enlarged tortuous right coronary artery (RCA) with a coronary sinus fistula complicated by tricuspid regurgitation.

## Case presentation

**Fig. 1:**
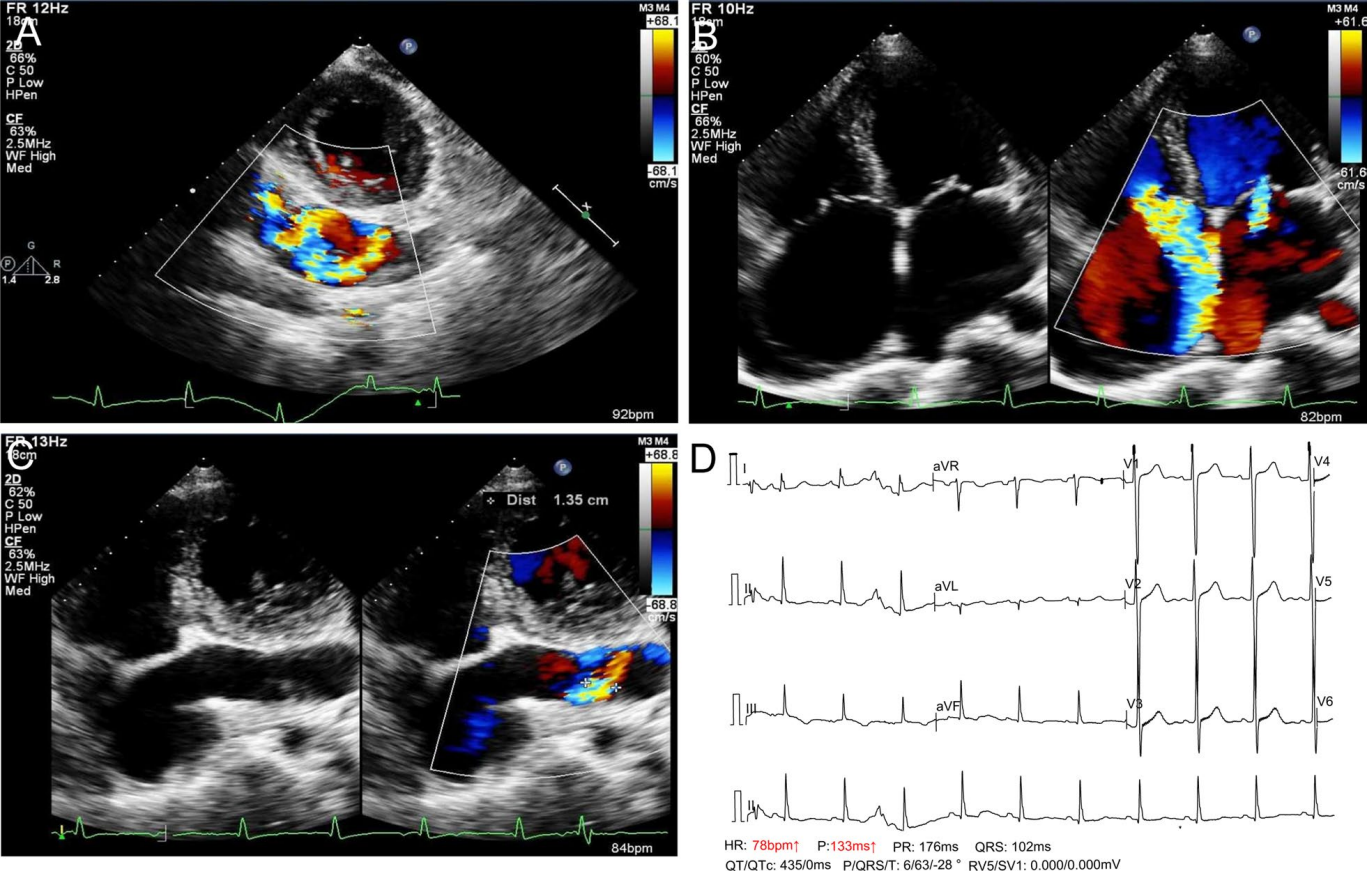
**A–C** Preoperative transthoracic cardiac ultrasound (TTE) showed severe tricuspid valve regurgitation, enlargement of right heart ventricle, moderate pulmonary artery systolic pressure, mild mitral valve regurgitation. Fistulous communication between the right coronary artery (RCA) and coronary sinus (CS) was associated with high pressure left-to-right shunt. **D** ECG showed no obvious sighs of myocardial ischemia

A 45 female was admitted to our hospital for tricuspid regurgitation due to large coronary artery to coronary sinus fistula. The patient experienced with exertional dyspnea, intermittent lower limbs and facial edema, no history of connective tissue diseases or chest trauma. Transthoracic cardiac ultrasound showed severe enlargement and tortuosity of right coronary artery due to congenital fistula to coronary sinuses, enlargement of right heart ventricle, severe tricuspid valve regurgitation (coaptation gap = 0.99 mm; CDFI: Vmax = 3.53 m/s, PGmax = 50 mmHg), moderate pulmonary artery systolic pressure (PASP:60 mmHg), mild mitral valve regurgitation (Fig. 1A–C). The blood flow velocity at right coronary artery ostium was 3.19 m/s, trans-ostium pressure was 41 mmHg. The RCA fistulized into the coronary sinus, 3.70 cm away from the orifice of coronary sinus. The blood flow velocity of orifice of coronary sinus was 2.78 m/s, trans-orifice of coronary sinus pressure was 31 mmHg. The ejection fraction of left ventricle was 50%. ECG showed no obvious sighs of myocardial ischemia (Fig. 1D).

**Fig. 2:**
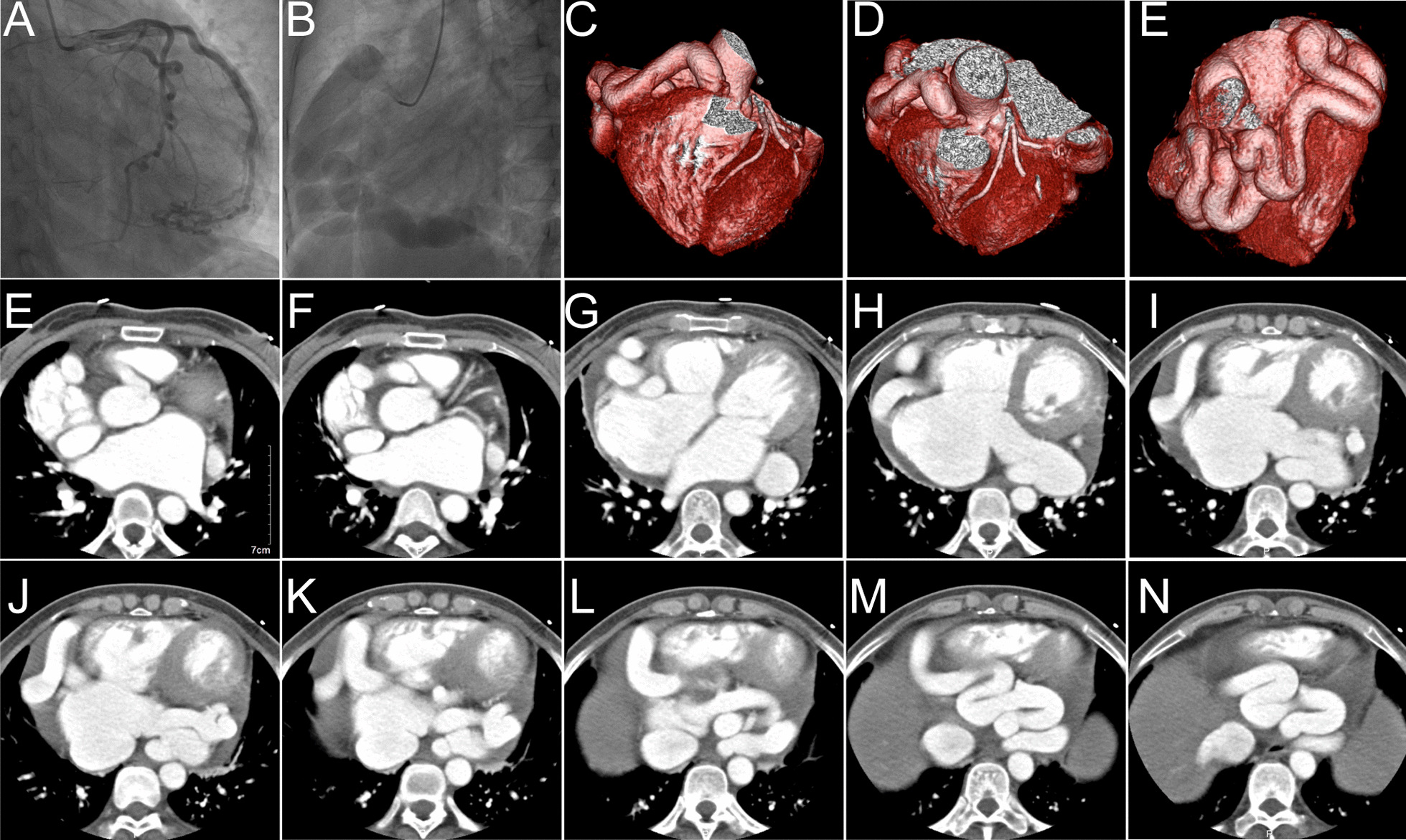
**A**, **B** Coronary angiography demonstrated normal left coronary artery blood flow and tortuously dilated right coronary artery. **C–N** Preoperative computed tomography angiography revealed that the whole course of RCA was severely dilated, tortuous RCA and abnormally fistulized to the posterior and inferior aspects of coronary sinus. The widest dimension of RCA was about 2 cm. Imaging findings also indicated the presence of persistent left superior vena cava (LPSV)

Coronary angiography demonstrated tortuously dilated right coronary artery and normal left coronary artery blood flow (Fig. 2A, B). Computed tomography angiography revealed that the whole course of RCA was dilated and abnormally fistulized to the posterior and inferior aspects of coronary sinus (Fig. 2C–N). The widest dimension of RCA was 2 cm.

**Fig. 3:**
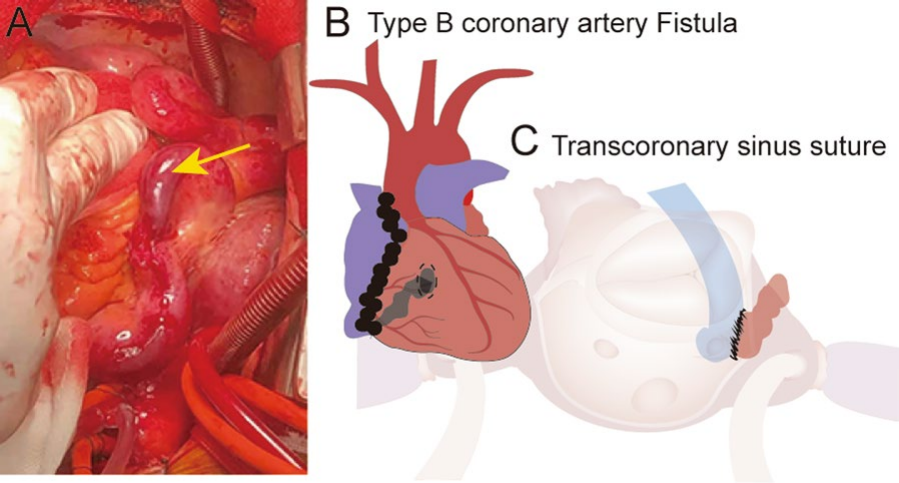
**A**, **B** After open the pericardium and under direct observation, we found the right coronary artery was apparently dilated and tortuous, fistulized to the posterior and inferior aspects of coronary sinus. **C** Diagram of the fistula repair. It was closed by continuously sutured directly with 5-0 prolene sutures

Based on the clinical information, a diagnosis of CAF was made. In consideration of the patient’s symptoms of right ventricular volume overload, surgery was indicated. Briefly, after routine median sternotomy, cardiopulmonary bypass was established by aortic and vena cava cannulation. Under direct observation, a large whole-course dilated, tortuous RCA on the surface of the heart was seen (Fig. 3A). Following aortic cross-clamping, perfusion of cold cardioplegic fluid, cardiac arrest was obtained. After open the right atrium, the fistula near coronary sinus orifice was observed, and was closed by continuously sutured directly with 5-0 Prolene sutures (Fig. 3B, C).

**Fig. 4:**
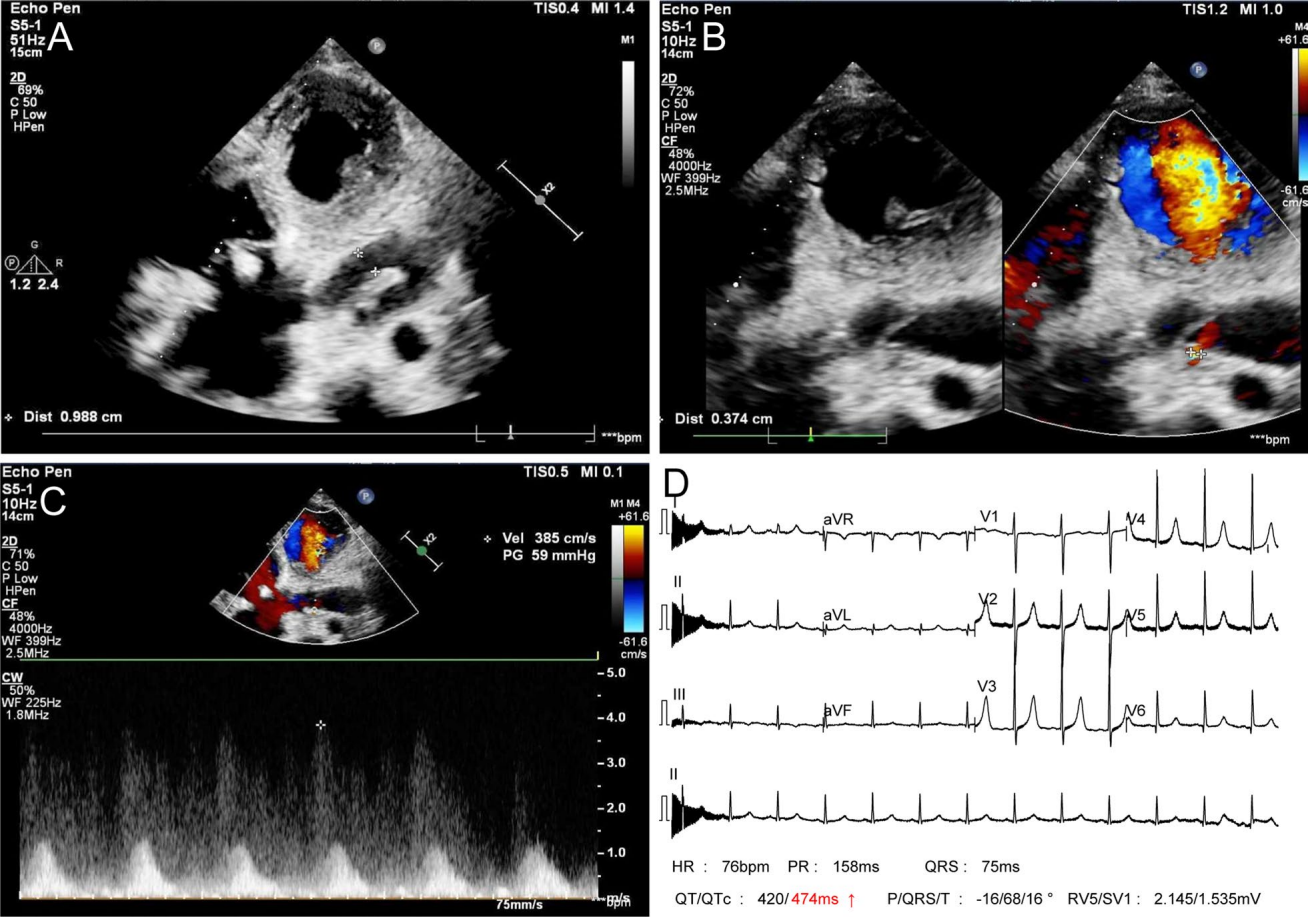
**A–C** Post-operative transthoracic cardiac ultrasound (TTE) showed mild tricuspid valve regurgitation, mild residual fistula. **D** ECG showed no obvious sighs of myocardial ischemia

**Fig. 5:**
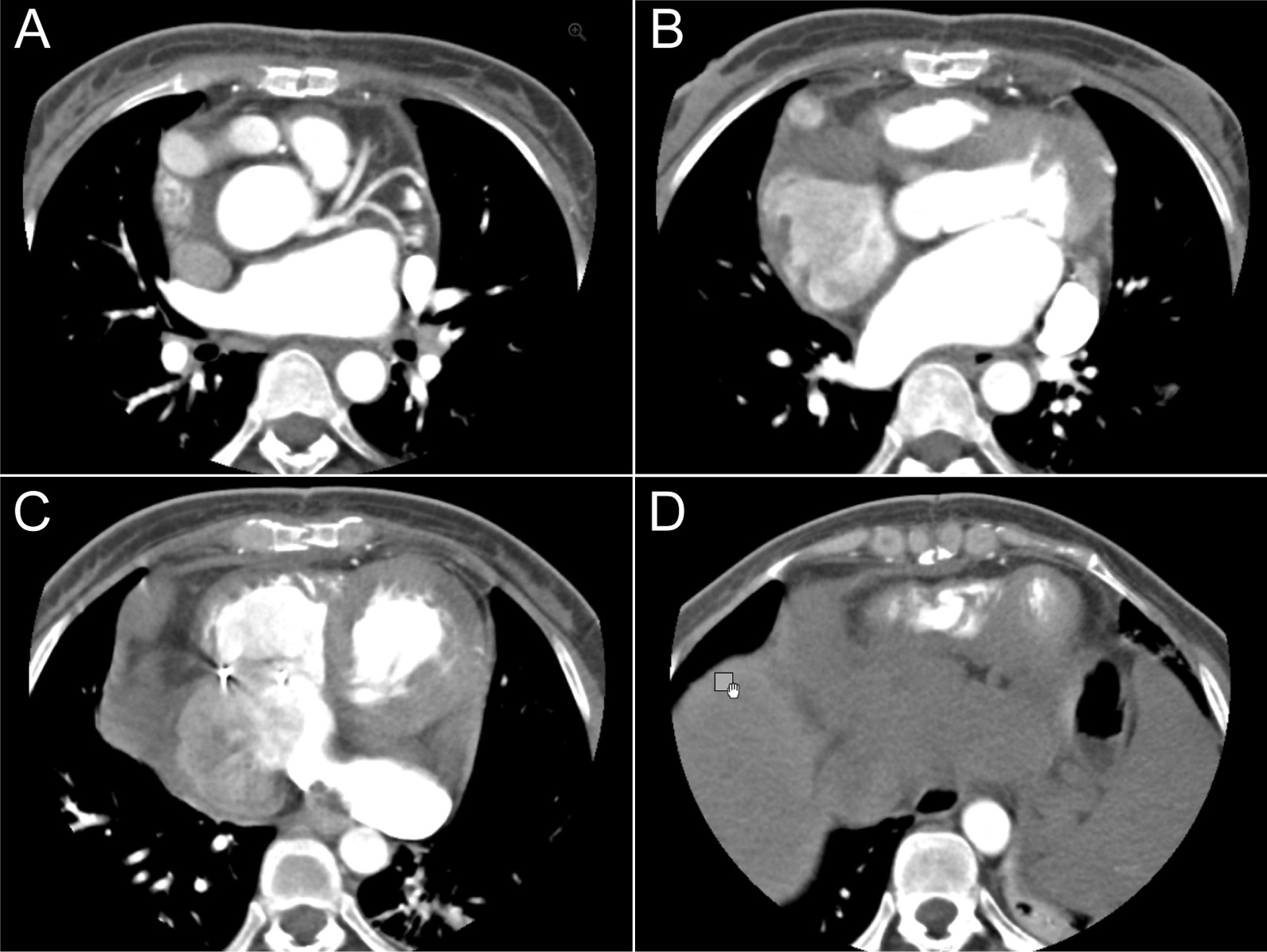
After one month fellow up, the coronary artery is not occluded, the diameter of coronary artery decreased slightly, but the velocity of blood flow in the distal coronary artery became slow

Postoperative transthoracic echocardiography showed mild residual fistula (Fig. 4A–C). ECG showed no obvious sighs of myocardial ischemia (Fig. 4D). The blood flow velocity of right main coronary artery was 0.85 m/s, pressure was 3 mmHg. The blood flow velocity at orifice of residual fistula was 3.85 m/s, trans-orifice pressure was 59 mmHg. The patient recovered uneventfully and was discharged. The patient was given oral warfarin anticoagulant therapy. At one month follow-up, computed tomography angiography revealed that the blood flow of RCA became lower (Fig. 5A–D).

## Discussion

Coronary arteriovenous fistulas (CAFs) are rare malformation. It has variable communications to great vessels or heart chambers. They occur in 0.1–0.2% of patients undergoing coronary arteriography. Coronary artery fistula occurs most often in the right coronary artery, followed by left anterior descending artery [2]. Potential clinically symptoms include angina, fatigue, dyspnea, heart failure., arrhythmia, embolization, rupture, myocardial infarction, infective endocarditis, and sudden death, which depend on size of the fistula, degree of associated shunt and associated complications, Due to the lack of specific signs and symptoms, its diagnosis mainly depends on imaging analysis. Coronary angiography is the golden standard to diagnose CAF, however, it sometime failed to demonstrate the origin, pathway, and outflow of fistula. Computed tomography angiography has been used complementary to demonstrate the complex anatomy of the fistula.

Since introduced by Krause in 1865, there is no consensus to the optimal treatment for CAFs. CAFs were classified angiographically into proximal (type A) or distal types (type B), which is helpful in considering the optimal surgical approach [3]. If the fistula was located at proximal segment of the coronary artery, direct epicardial ligation of the fistula may be optimal. Otherwise, the fistula was oriented at distal segment of the coronary artery, intracameral purse-string sutures at the site of termination with CPB or coronary artery graft bypass was needed. Moreover, the type B needs pay attention to slowing flow of coronary circulation or residual fistula and coronary dilatation after repair. Based on current evidence, the long-term survival was optimistic [4].

Unlike previous reported [5], in our case, the patient was found have a persistent LSVC. The presence of an LSVC increase the potential for brain abscesses and emboli due to intermittent right-to-left shunting, also cause procedural difficulties. Oxygenated blood returning from the lungs shunted into the CS through a high-pressure communication, producing significant volume overload, which lead progressive dilatation of tricuspid annuli, with consequent severe TR. Myocardial infarction (MI) after surgical ligation of CAF is a real concern and can occur early or late after surgical management (the risk is about 3.6%). The blood flow velocity of the right coronary artery decreased after the patient’s operation. Antithrombotic therapy is needed for such higher risk case. Residual small shunt may raise the possibility to reduce the risk of post operated thrombosis, but might exacerbate tricuspid valve regurgitation. Careful evaluation of tricuspid valve function should be needed during the long-term follow up. Since the patient is young, it is reasonable that interventional closure of residual fistula after carefully evaluation, for avoiding long-term complications of coronary sinus residual fistula: MI, thrombosis, arrhythmias, cardiomyopathy [6].

## Conclusion

We describe the case of a female who underwent successful surgical treatment of a severely enlarged tortuous right coronary artery (RCA) with a fistula draining into the coronary sinus (CS) complicated by tricuspid regurgitation. The management of CAFs should be individualized based on the features and associated symptoms of the CAF.

## Data Availability

Not applicable.

## Abbreviations

RCA: Right coronary artery
RC: Right coronary
CAF: Coronary artery to coronary sinus fistula
ECG: Electrocardiogram
CPB: Cardiopulmonary bypass
CS: Coronary sinus
TR: Tricuspid regurgitation
MI: Myocardial infarction

## References

[1] Majidi M, Shahzamani M, Mirhoseini M (2011). Clinical features of coronary artery fistula. J Tehran Heart Cent J Tehran Heart Cent.

[2] Ata Y, Turk T, Bicer M, Yalcin M, Ata F, Yavuz S (2009). Coronary arteriovenous fistulas in the adults: natural history and management strategies. J Cardiothorac Surg.

[3] Dimitrakakis G, Otto von Oppell U, eComment (2011). Surgical treatment of coronary arteriovenous fistulas. Interact Cardiovasc Thorac Surg.

[4] Said SM, Burkhart HM, Schaff HV, Connolly HM, Phillips SD, Suri RM, Eidem B, Rihal CS, Dearani JA (2013). Late outcome of repair of congenital coronary artery fistulas–a word of caution. J Thorac Cardiovasc Surg.

[5] Mitropoulos F, Samanidis G, Kalogris P, Michalis A (2011). Tortuous right coronary artery to coronary sinus fistula. Interact Cardiovasc Thorac Surg.

[6] Tang L, Wang ZJ, Tang JJ, Fang ZF, Hu XQ, Tai S (2020). Transcatheter closure of large coronary-cameral fistulas using the patent ductus arteriosus occluder or amplatzer vascular plugs. Int Heart J.

